# A prospective pilot study on MRI visibility of iron oxide-impregnated polyvinylidene fluoride mesh after ventral rectopexy

**DOI:** 10.1007/s10151-019-02022-w

**Published:** 2019-07-03

**Authors:** K. E. Laitakari, J. K. Mäkelä-Kaikkonen, E. Pääkkö, P. Ohtonen, T. T. Rautio

**Affiliations:** 1 Division of Gastroenterology, Department of Surgery, University Hospital of Oulu, Oulu, Finland; 2 Department of Radiology, University Hospital of Oulu, Oulu, Finland; 3 Medical Research Center Oulu, University of Oulu, Oulu, Finland

**Keywords:** Rectal prolapse, Ventral rectopexy, Mesh, Magnetic resonance imaging (MRI), MR contrasting implant, Iron oxide-impregnated polyvinylidene fluoride

## Abstract

**Background:**

Magnetic resonance imaging (MRI) provides excellent information about pelvic anatomy after ventral rectopexy, but the position of the conventional mesh is not seen constantly. Iron oxide-impregnated polyvinylidene fluoride (PVDF) meshes are proven to have MRI visibility in hernia or vaginal reconstructive surgery. This prospective pilot study was designed to assess the visualization, position, and shape of the magnetic resonance (MR)–visible synthetic pelvic mesh used in minimally invasive ventral rectopexy.

**Methods:**

Eight patients with pelvic organ prolapse were recruited for laparoscopic (LVMR) or robotic-assisted ventral mesh rectopexy (RVMR) with a synthetic MR–visible PVDF mesh. A follow-up visit was scheduled at 3 months after surgery. MR imaging was performed to evaluate the position and dimensions of the mesh and anatomical result. The visibility of the mesh in each sequence was assessed subjectively.

**Results:**

The visibility of the mesh was best on T1-weighted flash images. The mesh was also well visualized on T2-weighted sagittal images. T2-weighted images, in general, provided best visualization of the surrounding anatomical structures and enabled assessment of the mesh fixation.

**Conclusions:**

T2 sagittal and T1-weighted flash images provide the best information about the position and integrity of the iron oxide-impregnated PVDF mesh after LVMR or RVMR with a short examination time.

## Introduction

Laparoscopic ventral mesh rectopexy (LVMR) introduced by Andre D’Hoore has become commonly used treatment for rectal prolapse [1–4]. This minimally invasive procedure, also feasible with a robotic approach [5], offers the advantages of decreased risk of damaging autonomic nerves, recurrence, and new-onset post-operative symptoms. Anatomical and functional changes in the pelvic floor after anterior rectopexy have been described [6–8], but there are no studies that have investigated the position and dimensions of the mesh.

The risk of mesh-related complications like erosions, mesh infection, dyspareunia or fistula formation is quite low [8–10]. However, post-operative de-novo symptoms such as urinary retention, fecal incontinence, constipation or pelvic/abdominal pain are not so infrequent [4, 8, 9, 11]. The overall recurrence rate of rectal prolapse after laparoscopic or robotic-assisted ventral mesh rectopexy (RVMR) is up to 15.4% [4–6, 8, 11]. MR-contrasting implants have been suggested to be helpful in diagnosing post-operative problems non-invasively.

The aim of this prospective pilot study was to assess the visualization, position, and dimensions of the MR–visible synthetic mesh after LVRM or RVMR. The primary outcomes were the quantificational characterization of mesh position and anatomical changes.

## Materials and methods

### Study population and data collection

From February to April 2018, eight unselected consecutive patients with pelvic organ prolapse were recruited for laparoscopic or RVMR with a synthetic MR–visible polyvinylidene fluoride (PVDF) mesh in Oulu University Hospital, Finland. Written informed consent was obtained from all patients. All data about patient characteristics and post-operative recovery were collected prospectively. The study was approved by the local Ethics Committee.

### Surgical technique

The surgical procedures were primarily carried out as described by D’Hoore and Penninckx [2] with minor modifications. The da Vinci Surgical System (Intuitive Surgical Inc., Sunnyvale, CA, USA) with five trocar placements and side docking was used to perform RVMR. The mesh was positioned as far distally as possible and sutured to the levator muscles and on the anterior rectal wall with multiple interrupted seromuscular non-absorbable sutures (2-0 Ethibond, Ethicon Endosurgery). In laparoscopic procedures, only four to five sutures were used to fix the mesh on the proximal rectal wall, and the distal part of the mesh was fixed with glue. For the sacral promontory fixation, spiral attachments (Pro-Tack TM Fixation Device, Medtronic) were used. The peritoneum was closed over the mesh with continuous suture with absorbable V-Loc™ 90 (Medtronic). Peri-operative care was conducted according to the enhanced recovery after surgery protocol.

### Mesh information

MR–visible polyvinylidene fluoride (PVDF) 4 × 23 cm meshes (Dynamesh^®^ IPOM, FEG Textiltechnik, Aachen, Germany) containing paramagnetic iron oxide microparticles (Fe_3_O_4_ with iron load of 10 mg/g polymer) were used. This macroporous (> 1 mm) mesh consists of 88% visceral-sided PDVF monofilament and 12% parietal-sided polypropylene monofilament.

### Follow-up

A follow-up visit was scheduled at 3 months after surgery. Patients were evaluated for their pelvic clinical status and the functional results assessed with questionnaires reflecting quality of life and possible post-operative symptoms. MR imaging was performed for radiological evaluation of position and dimensions of the mesh and anatomical result.

### Magnetic resonance imaging

Magnetic resonance imaging (MRI) was performed by a 3 T magnet (Siemens, Vida, Erlangen, Germany). The patients were asked to empty the bladder before imaging. No other patient preparation was used. Patients were lying supine in the magnet. A body matrix surface coil was used in addition to the posterior spine coil.

T2-weighted sagittal, coronal, and transverse images were obtained (TR 3720–6100, TE 81–90, sagittal and coronal FOV 230, transverse FOV 200, 3 mm slice, 0.6 mm gap, sagittal and coronal matrix 256 × 320, transverse matrix 544 × 640). Breath-hold transverse T1-weighted vibe Dixon (TR 4, TE 1.3 and 2.5, FOV 309 × 380, 3 mm slice, 195 × 320 matrix) and T1-weighted (TR 129, TE 2.5, FOV 333 × 380, 3 mm slice, 210 × 320 matrix) flash images were also obtained. Total time of the examination was 30–35 min.

The visibility of the mesh in each sequence was assessed subjectively. Scores from 1 to 4 were used. Score 1 was given to image series if the mesh was visible in all slices. In score 2, 3, and 4, the visibility was ≥ 3/4, ≥ 1/2 or < 1/2 of the slices.

The position of the lower insertion point according to anorectal junction was assessed, as well as the insertion point to the levator muscle on each side. The length of insertion to the anterior rectal wall was measured. The width of the mesh was measured at the lower insertion point, at the highest insertion point in the rectum, and at the higher insertion point in the promontorium. Also, the narrowest part of the mesh was measured, as well as its distance from the highest rectal insertion point.

## Results

Eight female patients were included in this analysis, and their baseline clinical characteristics and used surgical technique are given in Table 1. All operations were primary except one robotic re-rectopexy for patient D (Table 1) with recurrent enterocele after previous ventral rectopexy. Mean operative time was 131 min (SD 44.4) and blood loss was 68 ml (SD 138.5). One laparoscopic operation was converted to open, and patient G (Table 1) had mild post-operative acute myocardial infarction. There were no peri-operative or any post-operative surgical complications. Mean hospital stay was 1.5 days (SD 0.76).

**Table 1:** Baseline characteristics and peri-operative outcome

Patient	Age (years)	ASA	BMI (kg/m^2^)	Diagnosis	Indication	Surgical technique	Operation time (min)	Blood loss (ml)
A	63	3	24	Enterocele	Incontinence	RVMR	86	20
B	56	2	22	Enterocele	ODS	RVMR	90	400
C	88	3	22	Prolapse	Prolapse	RVMR	121	0
D	53	2	23	Enterocele	Incontinence	RVMR^a^	174	0
E	77	3	27	Prolapse	Prolapse	RVMR	214	100
F	49	2	24	Enterocele	Incontinence	LVMR	148	0
G	77	3	25	Invagination	Incontinence	LVMR^b^	118	20
H	41	2	22	Invagination	Incontinence	LVMR	106	0

*ASA* American Society of Anesthesiologists, *BMI* body mass index, *ODS* obstructed defecation syndrome, *RVMR* robotic ventral rectopexy, *LVMR* laparoscopic ventral rectopexy

^a^Re-rectopexy

^b^Conversion

**Fig. 1:**
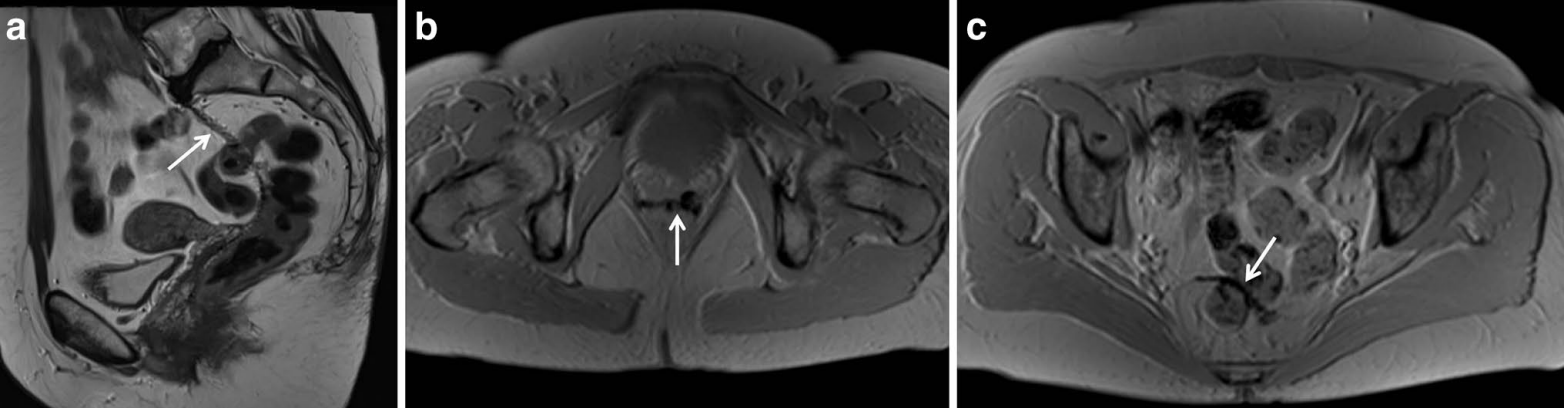
**a** The mesh is visualized in its full length on T2-weighted sagittal image. On T1-weighted flash images, the mesh is also well visualized close to the lower insertion point (**b**) and at the higher insertion on the rectal wall (**c**)

All patients had a 3-month follow-up with MRI imaging. The results of mesh position and dimensions are summarized in Table 2. The anatomical correction of the pelvic floor was excellent in all cases and there were no significant differences in any proportions of the meshes. Fixation of the meshes to the levator muscles and to promontorium was also seen (Fig. 1b; Table 2).

**Table 2:** Pelvic floor area measurements on MRI

Patient	Parameter								
	Anorectal junction^a^	Right levator muscle^b^	Left levator muscle^c^	Anterior rectal wall^d^	Lower insertion point^e^	Highest insertion point^f^	Narrowest part^g^	Distance from the insertion point^h^	Promontorium^i^
A	0	Yes	Yes	93	36	28	16	6	22
B	0	Yes	Yes	86	31	23	15	5	20
C	0	Yes	Yes	87	40	33	17	20	26
D	0	Yes	No	105	40	45	19	15	34
E	20	Yes	Yes	43	34	22	10	42	22
F	0	Yes	Yes	79	45	29	28	14	26
G	0	Yes	Yes	96	38	30	15	32	38
H	7	Yes	Yes	58	43	29	11	26	33

*MRI* magnetic resonance imaging

^a^Distance of the lower insertion point from the anorectal junction (mm)

^b^The position of the insertion point to the right levator muscle

^c^The position of the insertion point to the left levator muscle

^d^Length of insertion to the anterior rectal wall (mm)

^e^Width of the mesh at the lower insertion point (mm)

^f^Width of the mesh at the highest insertion point in the rectum (mm)

^g^Narrowest part of the mesh (mm)

^h^The distance of the narrowest part of the mesh from the highest rectal insertion point (mm)

^i^Width of the mesh at the higher insertion point in the promontorium (mm)

The visibility of the mesh was best on T1-weighted flash images (scoring 1 in all cases) as shown in Table 3. The mesh was also well visualized on T2-weighted sagittal images (scoring 1 in six cases, 3 in one case, and 4 in one case with severe movement artifacts). T2-weighted images, in general, provided best visualization of the surrounding anatomical structures (Fig. 1).

**Table 3:** The visibility of the mesh in MRI sequences

Patient	MRI sequence					
	T2 sagittal	T2 transverse	T2 coronal	T1 flash	Dixon in phase	Dixon out of phase
A	1	4	4	1	2	4
B	3	4	4	1	2	4
C	1	3	4	1	1	4
D	4	4	4	1	2	4
E	1	3	3	1	1	4
F	1	4	4	1	2	4
G	1	2	3	1	2	4
H	1	3	3	1	2	4

1, mesh was visible in all slices; 2, visibility was ≥ 3/4 of the slices; 3, visibility was ≥ 1/2 of the slices; 4, visibility was < 1/2 of the slices

*MRI* magnetic resonance imaging

## Discussion

This pilot study was designed to find the best way to visualize the iron oxide-impregnated PVDF meshes implanted for rectal prolapse. Our results showed that position and dimensions of this new mesh are seen sufficiently to make measuring using post-operative MRI. This would be useful in cases of post-operative mesh-related complications and recurrent symptoms; particularly, when planning reoperation to check if the mesh has gotten detached from the pelvic floor or promontory attachments.

There are few previous results showing the feasibility of iron oxide-impregnated PVDF meshes in hernia and vaginal reconstructive surgery [12–15]. However, this is the first study to demonstrate how the MR–visible synthetic mesh is seen after LVMR or RVMR in post-operative MRI. Therefore, it is impossible to compare our results to any previous data. We hope that other study groups will get interested in doing further research on iron oxide-impregnated PVDF meshes.

If the position and integrity of the mesh are in question, T2 sagittal and T1-weighted flash images would provide sufficient information with a short examination time. However, if there are other post-operative concerns, such as infection, a wider selection of sequences should be used together with intravenous contrast agent. Acquired information helps the design of future studies comparing different ventral rectopexy techniques, and especially mesh fixation alternatives.

## Conclusions

T2 sagittal and T1-weighted flash images provide the best information about the position and integrity of the iron oxide-impregnated PVDF mesh after LVMR or RVMR with a short examination time.
